# Frontiers of molecular crystal structure prediction for pharmaceuticals and functional organic materials

**DOI:** 10.1039/d3sc03903j

**Published:** 2023-11-03

**Authors:** Gregory J. O. Beran

**Affiliations:** a Department of Chemistry, University of California Riverside Riverside CA 92521 USA gregory.beran@ucr.edu

## Abstract

The reliability of organic molecular crystal structure prediction has improved tremendously in recent years. Crystal structure predictions for small, mostly rigid molecules are quickly becoming routine. Structure predictions for larger, highly flexible molecules are more challenging, but their crystal structures can also now be predicted with increasing rates of success. These advances are ushering in a new era where crystal structure prediction drives the experimental discovery of new solid forms. After briefly discussing the computational methods that enable successful crystal structure prediction, this perspective presents case studies from the literature that demonstrate how state-of-the-art crystal structure prediction can transform how scientists approach problems involving the organic solid state. Applications to pharmaceuticals, porous organic materials, photomechanical crystals, organic semi-conductors, and nuclear magnetic resonance crystallography are included. Finally, efforts to improve our understanding of which predicted crystal structures can actually be produced experimentally and other outstanding challenges are discussed.

## Abstract 2

Molecular crystal structure prediction has matured to the point where it can routinely facilitate the discovery and design of new organic materials.

## Graphical abstract

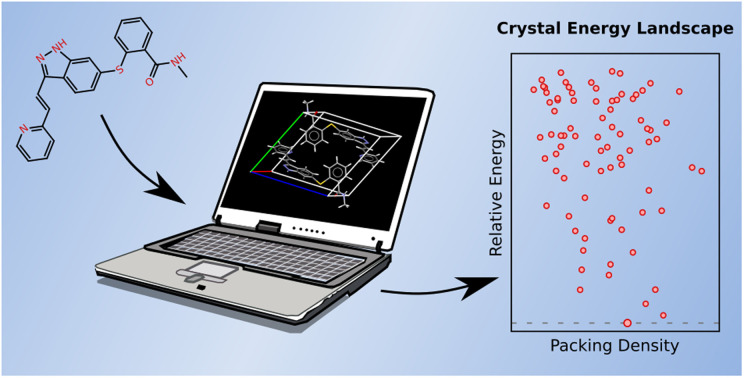

## 1 Introduction

Molecular organic crystals occur across many areas of chemistry. The majority of small-molecule pharmaceuticals are administered in crystalline form.^1^ Molecular crystals are key components of fertilizers,^2,3^ pesticides,^4,5^ and pigments.^6^ They can function as field effect transistors, light-emitting diodes, and photovoltaic cells.^7^ Porous organic crystals can perform gas storage and separations.^8^ Crystalline order can enable highly-selective solid-state syntheses,^9^ while crystalline phase transitions and solid-state chemical reactions create the basis for new mechanically-responsive “dynamic” materials.^10–12^

The properties and functions of these crystals, including color, stability, solubility, carrier mobility, *etc.*, often depend strongly on the crystal packing. Notably, about half of all organic molecules are thought to exhibit polymorphism,^13^ or the ability to adopt multiple distinct crystal packing motifs, and this creates both challenges and opportunities when working with organic materials. While the crystallization of the “wrong” polymorph can hinder the bioavailability of a pharmaceutical and force its recall, for example, the possibility to tailor crystal packing to achieve desired physical properties is alluring. Unfortunately, experimental polymorph control can be difficult, and even seemingly minor changes in the crystallization conditions or to the molecular structure can alter the crystal structure significantly. The choice of solvent system, heat, pressure, or time can similarly transform a system from one polymorph to another.

For these reasons, developing new organic materials often requires an understanding of the landscape of crystal structures that can occur for the species of interest. Given the difficulties in ensuring that all important crystal forms have been discovered experimentally, researchers have long sought the complementary ability to predict crystal polymorphs theoretically. Seventy years ago, science fiction author Robert Heinlein dreamed of a future when “mathematical chemists will design new materials, predict their properties, and tell engineers how to make them—without entering the laboratory.”^14^ Progress toward this goal remained slow for decades, and in 1998 Maddox famously referred to the difficulty in predicting crystal structures as “one of the continuing scandals in the physical sciences.”^15^ Since then, however, crystal structure prediction (CSP) has transformed from scandal to reality, and Heinlein's vision is finally now being realized for organic crystals.

Successful predictions continue to mount in recent Blind Tests of Crystal Structure Prediction which have been held every few years since 1999.^16–21^ The results of the most recent 7th Blind Test will be published in the near future. The scope of successful predictions has progressed from small, rigid molecules to larger pharmaceutical-sized molecules with conformational flexibility and/or disorder, and from single-component crystals to multi-component hydrates, solvates, co-crystals, and salts. Even the definition of what constitutes a “successful” crystal structure prediction has evolved to become more stringent over time. In the first Blind Test, for example, simply finding the experimental crystal structure during the search procedure was considered a partial success, even if the energy model ranked it poorly. Today, a successful CSP is expected to predict both the structures and the relative stabilities accurately, and sometimes also how those stabilities vary with temperature and pressure.

Thanks to this progress, the pharmaceutical industry is rapidly adopting CSP to help de-risk against the unexpected appearance of new crystal forms or to narrow the search space of crystal co-formers to be screened experimentally.^22–31^ CSP has expanded from a purely academic endeavor to one with multiple private companies developing software, creating new algorithms, and providing contract CSP services. Some larger pharmaceutical companies have their own internal CSP teams as well. Beyond pharmaceuticals, CSP is being used to understand or discover new functional organic materials. In all of these application areas, CSP is helping to solve difficult crystal structures, anticipating new crystal forms, guiding experimental researchers toward the discovery of those forms, and enabling rational materials design.^23,32–34^

This perspective article seeks to highlight what organic CSP can accomplish today, how it can transform the discovery and understanding for a broad range of problems in organic materials, and where major outstanding challenges remain. Section 2 discusses reasons why CSP is such a difficult problem, while Section 3 provides a high-level overview of the methods currently used to overcome those challenges. Section 4 presents a variety of recent case studies that highlight the diverse frontiers of CSP, including examples from pharmaceutical formulation, its incorporation into nuclear magnetic resonance (NMR) crystallography, the discovery of new, highly porous organic crystals, the study of photochemical transformations in the solid state, and efforts towards the rational design of new materials. Finally, Section 5 discusses several directions in the field that will likely prove important in the next few years. For further reading, readers are also referred to several excellent earlier reviews that focus on CSP methods and applications in greater technical detail.^22,23,34–44^

## 2 The crystal structure prediction challenge

**Fig. 1:**
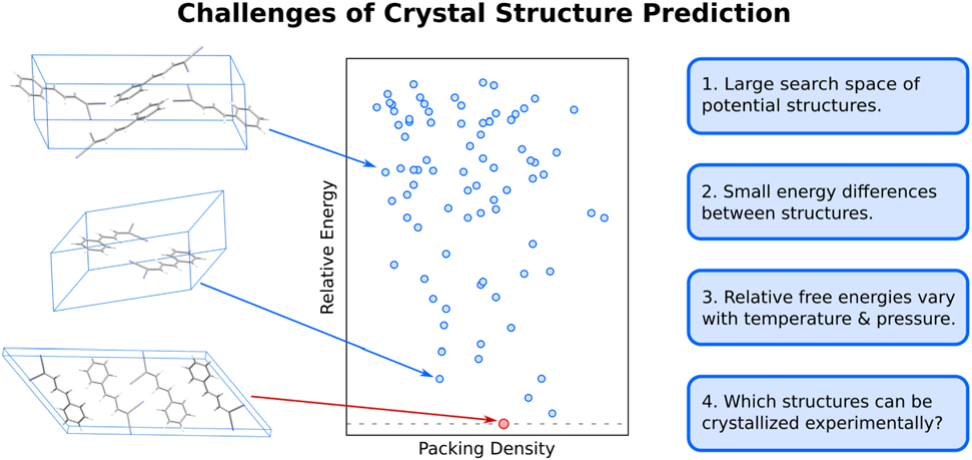
Organic molecular crystal structure is difficult due to the large search space of potential structures (blue dots) on the 0 K crystal energy landscape which are separated by small lattice energy differences. Moreover, the relative free energies between polymorphs vary as a function of temperature and pressure, and not all thermodynamically feasible crystal structures can be readily crystallized experimentally.

The difficulty of crystal structure prediction stems from several factors (Fig. 1): first, the search space of potential structures is massive, including 230 possible space groups, one or more molecules in the asymmetric unit, and, for many species, a competition between intramolecular conformational and intermolecular packing forces. While some of these complexities can reasonably be managed by, for example, constraining the search to the most common space groups and/or to crystals with just one molecule in the asymmetric unit, plenty of experimental crystals lie outside these constraints. Moreover, the conformational degrees of freedom in many modern active pharmaceutical ingredients and other highly flexible molecules are harder to circumvent, and they can dramatically increase the search space and the resulting computational costs of the structure prediction.

Second, the energy differences separating crystal polymorphs are small. Nearly all experimentally-known crystal polymorphs lie within 10 kJ mol^−1^ of one another,^13,45^ and the energy differences are often just ∼1–2 kJ mol^−1^. Those small energy differences manifest from competitions among the hydrogen bonding, electrostatics, induction/polarization, and van der Waals dispersion interactions within and between the molecules. Achieving kJ mol^−1^ resolution in modeling these diverse interactions can be difficult for both force fields and electronic structure methods, especially for conformational polymorphs^46^ whose crystal structures result from the interplay between changes in intramolecular conformation and the intermolecular crystal packing.

Third, while CSP often focuses on predicting 0 K crystal lattice energies,^40^ real-world crystal structures are determined by free energies at finite temperatures and pressures. In smaller molecules with limited flexibility, the differences between relative lattice energies and relative room-temperature free energies are usually small (<2 kJ mol^−1^).^45,47^ However, the magnitude of the relative entropic/free energy contributions can increase significantly in large, flexible drug-like molecules^30,48^ and disordered crystals.^49,50^ Moreover, factors such as thermal expansion and dynamics can alter the finite-temperature crystal structures themselves. The magnitude of these effects is frequently modest, but not always.

Finally, the vast majority of CSP research has focused on the thermodynamic stability of the crystal, but polymorph crystallization is highly influenced by kinetics. CSP routinely predicts far more thermodynamically viable candidate structures than are ever observed experimentally. There are multiple reasons for this over-prediction of structures,^51^ but crystallization kinetics are one major reason that more candidate polymorphs are not found experimentally. While there have been important advances in modeling organic crystal polymorph nucleation and growth in recent years,^52–55^ the statistical mechanical sampling challenges and the need for accurate but computationally inexpensive potentials represent on-going hurdles to reliable prediction of crystallization kinetics. Moreover, CSP routinely focuses on infinite crystals, ignoring the surface energy contributions that depend on the size and shape of the finite crystallite. Surface energies can be relevant when considering the stability of nanocrystalline formulations or polar crystals, for example.^56–61^ More detailed discussion of these issues is beyond the scope of this article.

## 3 Current methods of crystal structure prediction

### 3.1 Overview of hierarchical crystal structure prediction

**Fig. 2:**
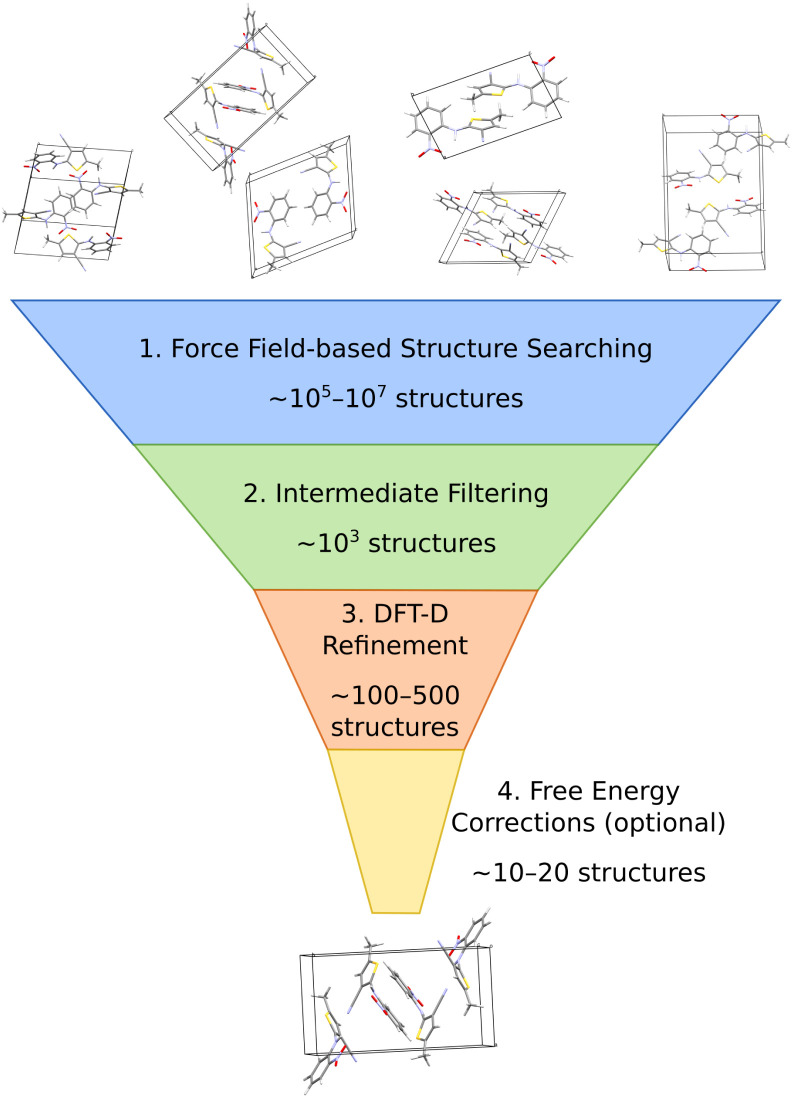
A typical hierarchical crystal structure prediction approach might (1) generate and rank large numbers of candidate structures with an inexpensive force field, (2) refine many of the most promising structures with some method of intermediate accuracy and computational cost, (3) perform dispersion-corrected DFT refinement on a few hundred structures, and (4) perhaps end with free energy calculations on a small number of structures.

The most common organic CSP approaches employ hierarchical stages of structure refinement and ranking (Fig. 2). For example, the first stage in the hierarchy might employ an inexpensive force field potential to screen ∼10^5^–10^7^ (pseudo-)randomly generated crystal structures, depending on the complexity of the species and the search space. The second-stage refines the ∼10^3^ lowest-energy structures with an intermediate-quality model. In the third stage, the few hundred most stable structures might then be refined further and ranked with dispersion-corrected density functional theory (referred to here as “DFT-D” for brevity, though many different dispersion-inclusive DFT models are used in practice). Optionally, one might perform final free-energy corrections for a handful of the most stable crystal structures to predict their stabilities at finite temperatures and pressures. More technical details have been reviewed elsewhere.^22,23,34–43^

A number of features factor into a successful crystal structure prediction. Ensuring a suitably-thorough search of crystal packing space is crucial. A routine search might focus only on crystals with a single molecule in the asymmetric unit (*Z*′ = 1) and from the ∼15–20 most common space groups that account for over 90% of observed organic crystals.^62^ More exhaustive searches might consider all 230 space groups and/or crystals with *Z*′ > 1. Significant additional complexity is introduced to the CSP for flexible molecules, due to the need to consider various equilibrium and non-equilibrium intramolecular conformations, or for multi-component crystals (co-crystals, solvates, hydrates, *etc.*), due to the much larger search space. Addressing these various complications can substantially increase the overall computational cost. Within the chosen search space, random^63–65^ or low-discrepancy pseudo-random search approaches^66–68^ are common in molecular CSP, though other global search algorithms such as simulated annealing,^69^ particle-swarm optimization,^70–72^ basin hopping^73^ or evolutionary optimization^42,74–77^ are also used.

The low-cost computational models used in the early stages of a hierarchical CSP enable broad searching, and the subsequent filtering out of poor candidates allows the more expensive methods to be applied only to the more promising candidates. Care must be taken to ensure the models used in the early stages are accurate enough to identify and select the relevant structures for later-stage refinement. For example, conventional off-the-shelf force fields are often not reliable enough for CSP. Section 3.2 will discuss how more customized potentials are often used instead.

Much of the current success in CSP stems from the widespread adoption of density functional theory for late-stage refinement and ranking. In the 4th Blind Test of CSP, a DFT-D-driven approach was the first to correctly predict the crystal structures of all the target molecules.^78^ The development of accurate, non-empirical, and computationally efficient van der Waals dispersion corrections for DFT,^79^ such as D3 and D4,^80–82^ many-body dispersion (MBD),^83–85^ and the exchange-hole dipole moment (XDM) model,^86^ has been particularly important. Generalized gradient approximation (GGA) density functionals used most frequently for computational expedience,^87–92^ though refining the single-point energies with hybrid density functionals improves the results meaningfully.^93–98^

While many CSP studies finalize their predictions with DFT-D structures and lattice energies, others proceed further to consider finite-temperature free energies. Surveys of small molecule crystals have found that vibrational free energy contributions change polymorph stability orderings for ∼10–20% of molecules at room temperature,^45,47^ though the differences between lattice energies and free energies can increase for larger, more complex systems due to conformational flexibility or disorder. For simpler molecules, harmonic, quasi-harmonic, and/or other simplified anharmonic treatments capture the vibrational free energy contributions reasonably well. On the other hand, molecular dynamics-based approaches are potentially superior for describing more complex crystals, assuming a suitably accurate potential energy model. Such techniques will be discussed further in Section 3.2. Overall, the combination of accurate DFT-D models and (sometimes) vibrational free energy contributions frequently leads to successful crystal structure predictions, as demonstrated for many Blind Test targets^91,92,94,95,97^ and for examples that will be discussed in Section 4.

In the end, performing a CSP produces a crystal energy landscape (Fig. 1), which is the set of predicted crystal structures and their relative lattice energies or free energies. Crystal energy landscapes at 0 K are often plotted as lattice energy *versus* crystal density, both because van der Waals forces generally favor more dense crystal packing motifs and because a scatter plot facilitates visualization of the large number of predicted structures. In some cases, one may simply wish to identify the most stable crystal structure(s). However, consideration of the full crystal energy landscape can provide valuable insights into the crystallization behaviors of a species^22,23^ or help elucidate crystal structure–property relationships for materials design. Before discussing such applications in Section 4, we discuss several areas where methodological developments are actively underway.

### 3.2 Areas of active methodological developments

#### 3.2.1 Improved models for early- and intermediate-stage structure refinement and ranking

In a hierarchical crystal structure prediction such as Fig. 2, the late-state DFT-D structure refinement and ranking typically consumes a large fraction of the total computational cost. Because the lower-cost intermediate stage models are generally less reliable than DFT-D, it is common to carry a relatively large number of structures forward to the DFT-D refinement to reduce the risk that an important structure is discarded early on (as happened in some cases during the sixth Blind Test^21^). Unfortunately, performing DFT-D refinement on many structures is computationally expensive. Therefore, the total computational cost of the CSP can potentially be reduced by improving the quality of the early/intermediate filtering model(s) so that fewer structures need to be carried forward to the DFT-D stage.

**Fig. 3:**
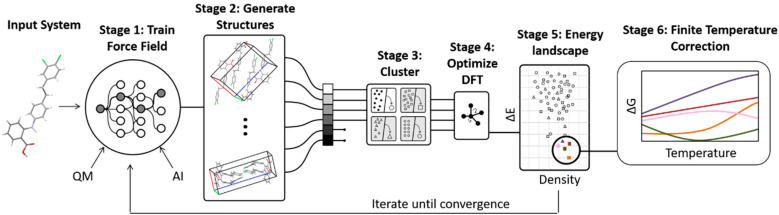
Some CSP procedures involve iterative cycles of force field fitting, structure prediction, and DFT-D structure refinement until the force field and DFT-D crystal energy landscapes are suitably consistent. Adapted with permission from ref. 48. Copyright 2020 American Chemical Society.

A number of strategies are currently being used to achieve this. One very successful approach involves parameterizing tailor-made force fields for each system based on DFT-D calculations.^99^ The force fields often employ fairly standard functional forms, with terms describing the intramolecular geometry, short-range intermolecular repulsion, long-range London dispersion, point-charge or multipolar electrostatics, and sometimes induction/polarization,

1 *U* = *U*_intra_ + *U*_rep_ + *U*_disp_ + *U*_es_ + *U*_pol_

but system-specific parameter tuning achieves higher accuracy than could typically be obtained with off-the-shelf force fields. Multiple force fields can be fitted to different subsets of data to predict and score structures independently, thereby potentially increasing the extent of the crystal packing space searched and providing insight into the uncertainties in the models.^72^ Moreover, as the CSP proceeds, the force fields can be reparameterized iteratively based on the results of DFT-D structure refinement as well as monomer/dimer quantum mechanical benchmarks (Fig. 3).^72^ Iterating the force field parameterization toward self-consistency with DFT-D helps ensure the search is performed with near-DFT-D quality. This iterative process also produces a more robust force field that can be used to evaluate finite-temperature free energy corrections.^48^ Machine learning potentials represent a natural extension of this idea.^44,100,101^

Low-cost semi-empirical methods are similarly promising for intermediate refinement of crystal structures and lattice energies.^102–109^ These can be further combined with Δ-ML, in which an ML model is trained to correct a simpler model up toward the quality of a more expensive one. Species-specific Δ-ML models have been used in CSP to correct semi-empirical density functional tight binding (DFTB) toward the accuracy of hybrid functional DFT-D,^110,111^ or to correct GGA functionals up to hybrid functional DFT-D or correlated wave function methods.^112,113^

Finally, *ab initio* force fields fitted to symmetry-adapted perturbation theory (SAPT)^114,115^ have also improved considerably. SAPT calculations naturally decompose the different types of intermolecular interactions (electrostatics, exchange-repulsion, *etc.*), which can be used to help ensure physically-sensible parameter fits in the potentials. Successful SAPT potentials could already be found in the literature 15 years ago,^116–120^ but the algorithms and protocols have now matured to enable highly-automated fitting for organic molecules with modest conformational flexibility.^121,122^ A recent study^123^ of fifteen organic molecules found that this approach placed the experimental structure within the top 10–20 structures (and often in top 5). Subsequent DFT-D refinement of the top 20 structures generated by these potentials for each species ranked the experimental structure as the most stable one in every case. Thus, these potentials are very accurate on their own and can provide an excellent short-list of candidate structures for subsequent refinement with fully quantum mechanical approaches. The biggest outstanding question is how efficiently these fitting algorithms can be generalized to highly flexible molecules

#### 3.2.2 Addressing DFT delocalization error in crystal structure prediction

Many CSP successes rely on dispersion-corrected DFT functionals. Commonly-used GGA and hybrid density functionals balance accuracy and computational cost and usually enable reliable refinement and ranking of hundreds of crystal structure candidates. However, approximate density functionals generally suffer from delocalization error (a.k.a. many-body self-interaction error),^124,125^ which manifests as a spurious tendency to prefer overly delocalized electron densities. Delocalization error leads to systematic errors such as the underestimation of band gaps, underestimation of chemical reaction barriers, erroneous spin state energy differences, over-estimation of hydrogen bond strengths, and problematic conformational energies.

**Fig. 4:**
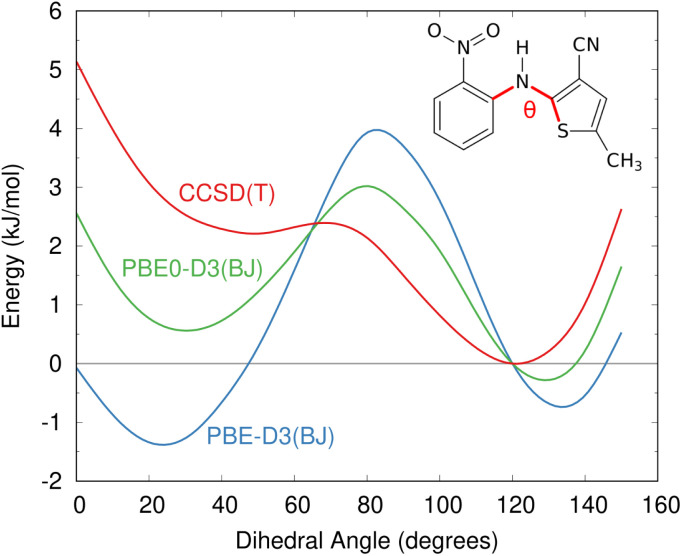
Delocalization error in GGA and hybrid functionals such as PBE and PBE0 leads to over-stabilization of more planar conformations of the ROY molecular relative to those with a dihedral angle closer to 90°, as compared to high-level coupled cluster benchmarks.^126^ This impacts the predicted crystal energy landscape, as will be discussed in Section 4.3.

The impacts of delocalization error in CSP were first highlighted by Johnson and co-workers in the context of reanalyzing the conformational energies in candidate structures for Blind Test molecule X,^92^ the lattice energies of halogen bonded crystals,^127^ and, most dramatically, by showing how it could spontaneously convert neutral acid-base co-crystals to their charged salt forms.^128^ The present author's group has since found many more examples where DFT delocalization error significantly impacts the relative stabilities for polymorphs of small molecules,^126,129,130^ pharmaceuticals,^126^ rubrene organic semi-conductor materials,^131^ and photochromic materials.^132–137^ All of these systems have crystal structures which differ in the extent of π conjugation, either due to changes in the intramolecular conformation (conformational polymorphism) or chemical reactions that convert sp^2^-hybridized atoms to sp^3^-hybridized ones (*e.g.* cycloaddition reactions). Fig. 4 shows how DFT delocalization error over-stabilizes the more planar conformations of the ROY molecule,^126,138,139^ the impacts of which will be discussed further in Section 4.3.

Delocalization error is particularly pronounced in GGA functionals such as PBE. Hybrid functionals such as PBE0 help mitigate the impacts of delocalization error,^96,97^ though the necessary amount of exact exchange needed can vary.^97,126^ Because the impacts of delocalization error on conformational energies are intramolecular in nature,^126,140^ an alternative strategy can be to perform a simple conformational energy correction,

2 *Ẽ*_crystal_ = *E*^DFT^_crystal_ − *E*^DFT^_intra_ + *E*^High^_intra_

that computes the DFT crystal energy *E*^DFT^_crystal_ and replaces the DFT-D intramolecular energy *E*^DFT^_intra_ with one computed using a more advanced model that is free of delocalization error, *E*^High^_intra_, such as correlated wave function methods^126^ advanced density functionals, or even density-corrected DFT.^141^

#### 3.2.3 Improved treatment of finite-temperature free energies

Switching the focus from 0 K lattice energies (*E*) to finite-temperature Gibbs (*G*) or Helmholtz (*F*) free-energies,

3 *G*(*T*,*P*) = *E* + *F*_vib_(*T*) + *PV*

can be important for making real-world predictions about the most stable polymorphs, polymorph phase transitions, the formation of hydrates as a function of humidity, *etc.* The simplest approximation for these effects involves computing the static harmonic Helmholtz vibrational free energy contributions *F*_vib_*via* lattice dynamics.

The quasi-harmonic approximation^142,143^ refines the treatment further by approximating how the phonons and *F*_vib_ contributions change as a function of unit cell volume, which is especially important for the low-frequency modes.^144^ The quasi-harmonic approximation enables predicting the temperature-dependent thermal expansion of the crystal lattice up to moderate temperatures, leading to improved-quality structures,^145–147^ thermochemical properties,^148–151^ spectroscopy,^152–154^ and even polymorph phase diagrams.^152,155,156^

Lattice dynamics calculations are considerably more expensive than computing the energy, particularly due to the need to capture phonon dispersion. For this reason, they are typically computed with relatively inexpensive DFT-D functionals. A multi-level approach that combines a higher-level treatment of the phonons in the crystallographic unit cell with a lower-cost treatment in the supercell can reduce the costs further.^157,158^ Although the quasi-harmonic approximation improves the description of lower-frequency modes, it does not address anharmonicities in the higher-frequency modes that are insensitive to the lattice parameters.^144^ One simple approach for those phonon modes employs a 1-D anharmonic model to improve the description of each individual mode.^94,95^ Vibrational self-consistent field calculations can capture anharmonicity more fully,^159,160^ albeit at significantly higher computational cost.

Alternatively, molecular dynamics (MD) techniques can improve upon these static lattice dynamics approaches. MD simulations naturally capture anharmonicities.^144^ Moreover, the finite-temperature dynamics will sometimes sample multiple minima on the potential energy surface, capturing contributions which would be missed entirely by (quasi-)harmonic models.^161,162^ MD approaches are also inherently better-suited to describing dynamically disordered crystals.

**Fig. 5:**
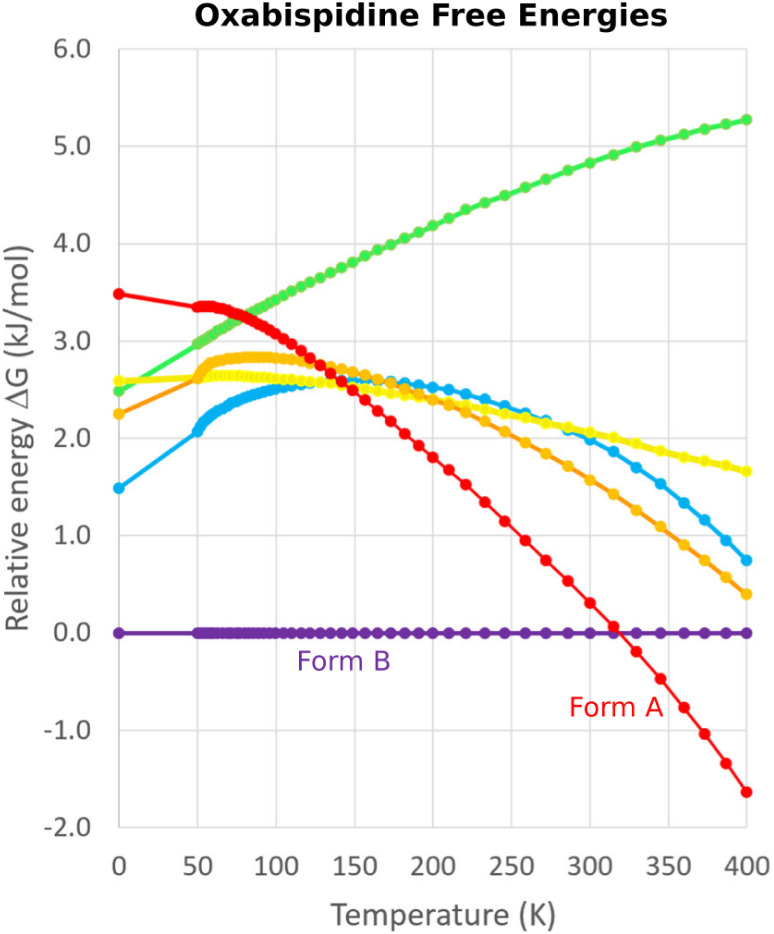
Free energy calculations on experimentally-known forms A and B of oxabispidine and several other predicted polymorphs find that form A only becomes the most stable form near room temperature. Adapted with permission from ref. 30. Copyright 2021 American Chemical Society.

One successful MD approach employs a pseudo-supercritical path approach to relate the free energies of the crystal polymorphs to that of an Einstein crystal reference state.^49,163,164^ For example, a CSP study of the polymorphs of drug candidate oxabispidine found that the form A was several kJ mol^−1^ less stable than form B, contrary to experimental observations. However, applying this free energy correction approach on top of the 0 K lattice energy predictions demonstrated enantiotropic relationship, with form A becoming the thermodynamically preferred form near ambient temperature (Fig. 5).^30^ Beyond classical molecular dynamics, path integral studies have shown that nuclear quantum effects can also be important for determining the relative polymorph stabilities in aspirin^165^ and ices.^166^

The biggest challenge with MD approaches is the need for extensive sampling, which means that *ab initio* MD simulations are extraordinarily expensive computationally—*e.g.* ∼2 million central processing unit (CPU) hours for paracetamol.^165^ Therefore, inexpensive energy potentials must be used in practice. As noted before, standard force fields will frequently lack the requisite accuracy needed for CSP applications. However, good-quality tailored force fields and machine learning potentials being fitted as part of the search process (as described above) can also be used for the free energy simulations.^48,101^ Re-weighting strategies that map from a low-cost free energy simulation to a higher-level one with only moderate sampling at the high level are also possible.^163,164^

MD free energy approaches have benefits beyond simply predicting polymorph stabilities. Molecular crystal free energy landscapes tend to be smoother than lattice energy ones, with multiple lattice energy minima separated by small barriers coalescing into a single free energy well at finite temperatures. This feature enables reducing the number of predicted structures on a crystal energy landscape or even searching for crystal structures directly on the free energy landscape (see Section 4.9).

## 4 Selected applications at the frontiers of crystal structure prediction

Having discussed some of the model features that lead to successful crystal structure prediction, we now focus on case studies that demonstrate the range and capabilities of present-day CSP. These examples were chosen to highlight the diverse ways in which CSP can complement experiment across a broad range of organic materials, rather than aiming for a comprehensive review of the literature.

### 4.1 Pharmaceutical solid-form screening: rotigotine and galunisertib

Choosing a suitable solid form for manufacturing is an important step in pharmaceutical formulation. Researchers desire crystals with suitable solubility profiles, mechanical properties, and stability. They want to avoid the surprise, late-stage appearance of new polymorphs with undesirable properties, such as those which necessitated the recall and reformulation of ritonavir^167,168^ and rotigotine.^169,170^ The risks are significant: it has been estimated that the most stable crystal form has not yet been discovered experimentally for some ∼15–45% of small-molecule pharmaceuticals.^28^ By providing a detailed understanding of the crystal energy landscape, CSP can complement experimental solid-form screening and help manage the risks of late-appearing polymorphs in pharmaceutical development.^22,23,171^

Consider two examples: rotigotine and galunisertib. Transdermal rotigotine patches are used to treat Parkinson's disease and restless leg syndrome. In 2008, the unexpected appearance of snowflake-like and highly insoluble crystals of a new crystal polymorph (form II) on the patches led to a major recall and restrictions on the drug in Europe, and its complete withdrawal from the U.S. market.^170^ It took four years to reformulate the patches and return them to the U.S. market.^169^

**Fig. 6:**
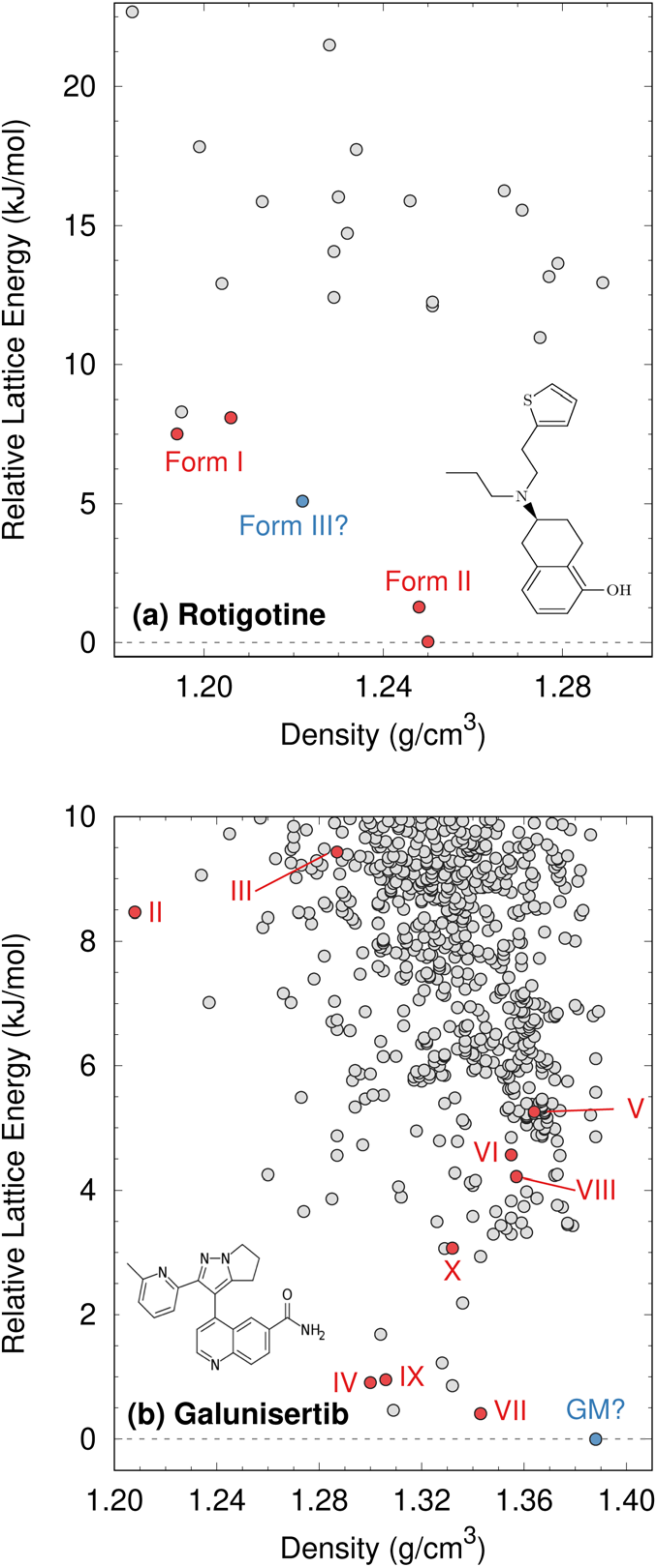
Predicted crystal energy landscapes for (a) rotigotine^172^ and (b) galunisertib.^26^ Red points indicate experimentally-observed polymorphs. For rotigotine, a pair of static structures was identified for each of forms I and II which correspond to the two possible conformations of the disordered thiophene ring. The structures labeled “form III” for rotigotine and “GM” for galunisertib have not yet been found experimentally.

Although CSP techniques were less mature in 2008, recent work by Mortazavi *et al.*^172^ demonstrates how modern-day CSP techniques could have anticipated form II rotigotine. Starting from only the 2-D molecular structure of rotigotine, they employed a mixture of tailor-made force fields (fitted against DFT-D calculations), dispersion-corrected DFT, and harmonic vibrational enthalpy/free energy contributions to predict the most stable crystal structures of rotigotine, including both forms I and II (Fig. 6a). Their models indicate that form II is 7.6 kJ mol^−1^ more stable than form I, in exceptional agreement with the 7.5 kJ mol^−1^ measured experimentally (such excellent agreement probably reflects some fortuitous error cancellation). Today, a CSP prediction of a new polymorph that was so much more stable than the known form would warrant significant concern and would motivate further experimental screening efforts. Moreover, the higher packing density predicted for form II also would suggest that high-pressure experiments might facilitate its crystallization. In fact, similar CSP insights motivated the high-pressure crystallization experiments that discovered new polymorphs of dalcetrapib^27^ and iproniazid,^171^ as will be discussed in Section 4.9.

Overall, rotigotine has a sparse crystal energy landscape, with only two predicted crystal structures other than forms I and II in the low-energy (10 kJ mol^−1^) region. One of those has stability intermediate between forms I and II. This putative “form III” has never been observed experimentally, and perhaps further investigations are warranted.

Whereas the CSP of rotigotine was performed long after its behavior was understood experimentally, CSP was directly integrated into the solid-form screening process for galunisertib.^26^ This drug candidate for metastatic malignant cancer^173^ has a complicated solid-form landscape: ten neat polymorphs and over 50 crystalline solvates have been discovered to-date. Its propensity for solvate formation complicated the experimental search for neat polymorphs, and a CSP was performed to identify any potentially important missing forms. The CSP revealed hundreds of potential crystal structures in the 10 kJ mol^−1^ energy window (Fig. 6b). Such densely populated crystal energy landscapes are unfortunately more typical for pharmaceuticals than the sparser rotigotine one.

The initial CSP for galunisertib predicted seven of the ten of the polymorphs eventually found experimentally, but it missed the remaining three due to search constraints that had been imposed to expedite the CSP. As crystal forms lying outside the initial CSP search space were discovered experimentally, a second, broader CSP was performed using techniques very similar to those for rotigotine. This second landscape successfully predicted all experimentally-discovered polymorphs.

The galunisertib CSPs helped solve the crystal structures of forms VII and VIII. Experimental difficulties obtaining pure crystals of these forms complicated the powder X-ray diffraction patterns, but the structures were eventually solved using comparisons against simulated powder diffraction patterns computed on candidate CSP structures. On the other hand, both CSPs predict that the most stable “global minimum” crystal structure has not yet been found experimentally, despite extensive efforts. This unrealized global minimum structure highlights two potential issues in CSP which will be discussed later: when are the accuracy limitations of widely-used DFT-D models problematic (Section 4.3)? When are predicted crystal structures actually crystallizable (Section 4.9)?

### 4.2 Addressing the further complexities in pharmaceutical crystal structure prediction

With mounting numbers of successful polymorph predictions for neat pharmaceuticals, CSP techniques are increasingly being applied to more complicated aspects of pharmaceutical formulation,^23^ including disorder and multi-component hydrates, solvates, and co-crystals. Cases such as the experimental cancer drug gandotinib,^25^ with its multiple hydrates, disorder, and difficulties crystallizing various forms exemplify the real-world complexities of pharmaceutical solid-form landscapes.

Consider first disorder, which is present in ∼20–25% of crystal structures.^50^ Static disorder results from molecules adopting a statistical distribution of different configurations or orientations in the lattice, while dynamic disorder is associated with the finite-temperature motions of molecules in the crystal. The distinction between the two types of disorder is not always sharp, however, and it can even vary with temperature.^174^ Both types of disorder can stabilize a crystal structure entropically. Typical CSP protocols neglect disorder, though the prediction of multiple closely-related crystal structures with similar lattice energies can be suggestive of a greater likelihood for disorder to occur in the experimental crystal structures.^175–177^

To obtain more quantitative results, disorder needs to incorporated into the models. Dynamic disorder can potentially be described *via* molecular dynamics simulations,^49,161,175^ for example, while a symmetry-adapted ensemble model which includes weighted energy contributions from all the configurationally unique structures is often used to treat crystals with static disorder.^178^ Such descriptions are considerably more computationally demanding than conventional static structure models, unfortunately. A symmetry-adapted ensemble for a system with *N* disordered sites having two possible states each requires evaluating the energy for 2^*N*^ possible configurations, though symmetry reduces the number of unique configurations in practice.

Accounting for the effects of disorder can be important. A CSP study on the antihistamine medication loratadine,^50^ for example, found multiple crystal structures corresponding to different components of the disorder. The initial landscape suggested that form I was relatively high in energy compared to other predicted forms. Form I became the most stable form only after it was modeled with a symmetry-adapted ensemble. Similarly, the initial predicted crystal energy landscape of gandotinib suggested that the most stable crystal polymorph had not yet been found experimentally. However, accounting for the disorder in form I made it isoenergetic to the predicted global minimum structure.^25^

Multi-component crystals are also extremely common in pharmaceuticals. Incorporation of water or other solvent molecules into a molecular crystal structure occurs frequently. In other cases, the active pharmaceutical ingredient (API) is deliberately crystallized as a salt (*e.g.* with hydrochloride) or with inactive co-formers to improve their solid form properties. CSP of multi-component systems can be considerably more difficult than single-component systems. Predicting when the co-crystal is thermodynamically preferred can be done pretty reliably.^179–181^ On the other hand, the presence of multiple species increases the crystal packing search space considerably,^182^ especially when multiple potential stoichiometries need to be considered.^183–185^

**Fig. 7:**
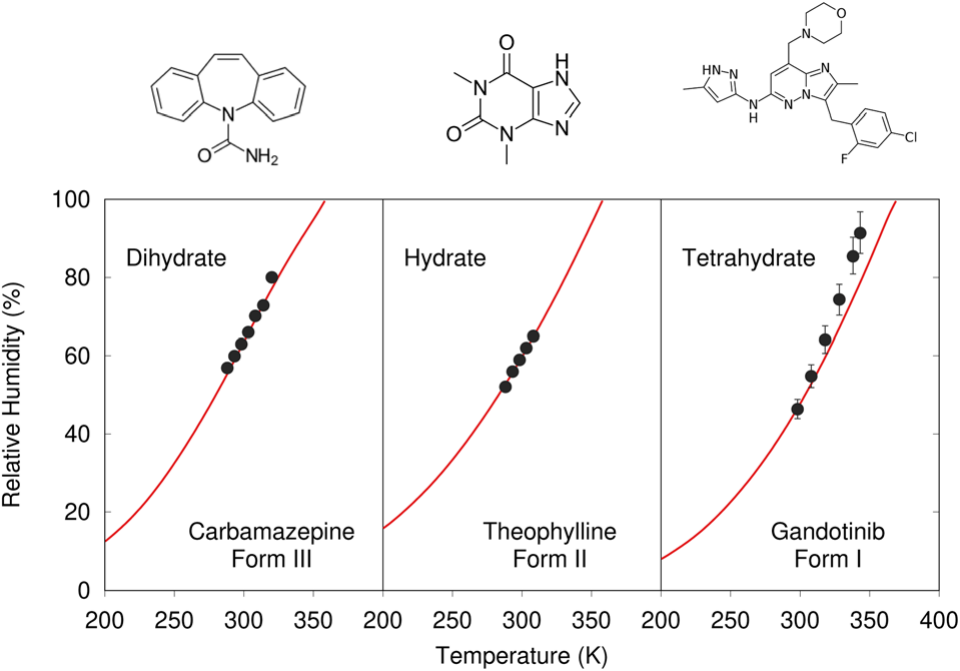
Predicted phase-boundary between hydrate and anhydrate forms of three drugs as a function of temperature and relative humidity. They show nearly quantitative agreement between theory (red lines) and experimentally-derived coexistence points.^186^

Despite these challenges, clear progress is being made. A number of successful hydrate predictions have been performed,^187,188^ including ones that predicted the correct stoichiometries.^185^ The use of free energy calculations to compare the stabilities of different co-crystal stoichiometries has also been demonstrated.^31^ Data-driven algorithms can identify plausible locations for water molecules within an anhydrous crystal structure, enabling a high-throughput screen of potential hydrates from an existing CSP landscape.^189^ Separately, Dybeck *et al.* impressively demonstrated that with the help of one experimentally determined co-existence point, the phase boundary between anhydrate and hydrate forms could be predicted as a function of temperature and relative humidity to within 10% relative humidity of experiment (Fig. 7).^186^ An example of co-crystal stoichiometry prediction was also included in the recent 7th Blind Test, with results to be published soon.

For an example of a successful CSP applied to a multi-component salt crystal, consider the recent studies of the sleep-related drug candidate B5.^190^ Whereas the neutral form of B5 has just one important crystal form,^190^ understanding the solid-form landscape of its hydrochloride salt B5HCl proved much more difficult.^177^ Extraordinary experimental effort was required to uncover two neat polymorphs of B5HCl, a dihydrate, and 11 alcohol solvates of B5HCl. The concurrent CSP study made several contributions to the eventual understanding: It highlighted the stability of form I, which helped explain its insolubility in various solvents and the difficulties in producing other crystal forms experimentally. It showed that the experimental forms discovered included examples of all major packing motifs found on the computational landscape, suggesting that the experimental screen was suitably complete. Moreover, the large number of closely related crystal structures on the computed landscape also pointed to the likelihood of disorder, especially for one particular conformation of the B5H^+^ molecule. This helped rationalize the experimentally-observed disorder and difficulty in growing crystals that were suitable for diffraction.

Finally, a typical solid-form screen might consider multiple different possible co-formers, potentially multiplying the number of CSPs that may need to be performed. Sugden and co-workers recently demonstrated one clever approach for simplifying this task.^191^ Their standard CSP approach employs pre-fitted local approximate potentials to describe important intramolecular conformational flexibility in their molecules,^192–194^ and generating those models from quantum mechanical calculations requires non-trivial effort. However, by creating a library of these conformational energy models for commonly-used co-former species in advance, they can quickly run a CSP for a given API with a whole suite of potential co-formers. After fitting the local approximate models for the new API, they can run a CSP to screen each API + co-former combination in just ∼2–3 days on a moderately sized cluster. Testing on three different drug molecules found that these relatively fast CSPs proved sufficiently accurate to rule out co-former candidates that were unlikely to form co-crystals experimentally, even if additional effort might be to refine the predictions for the most promising co-formers.

### 4.3 ROY and the impacts of DFT delocalization error

**Fig. 8:**
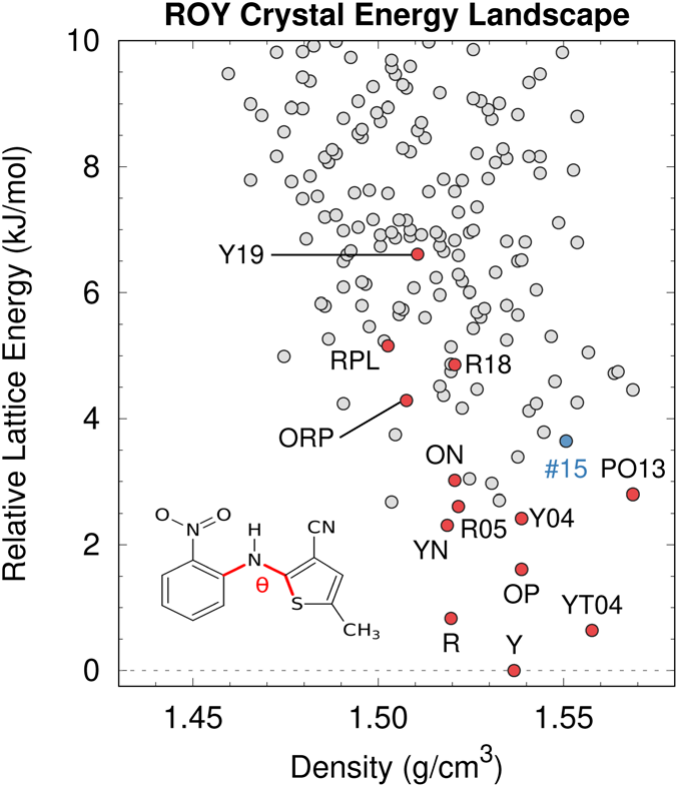
After addressing the DFT delocalization error issues, the predicted crystal energy landscape of ROY shows that the lowest-energy polymorphs have already been discovered experimentally (red). Interestingly, the hypothetical rank #15 structure in blue is predicted to become the most stable structure near 10 GPa. Figure adapted from ref. 129.

While CSP is increasingly successful, factors such as DFT delocalization error can still lead to incorrect predictions. The ROY molecule, so named for its vibrant red, orange, and yellow crystals,^195^ is a classic example of polymorphism and holds the current world record with 12 fully-characterized polymorphs,^196–204^ plus a thirteenth incompletely characterized form.^139,205^ Several ROY polymorphs were discovered/solved in the past few years.^200–204^ Despite the importance of this system to the field of polymorphism, predicted crystal energy landscapes of ROY were highly inconsistent with experimental polymorph stabilities rankings^139,200,206^ until very recently. The conformational flexibility of the ROY molecule (Fig. 8) is the primary factor behind ROY's colors^207–210^ and its propensity for polymorphism, but it also caused problems for CSP. As shown in Fig. 4, DFT delocalization error over-stabilizes the orange and red polymorphs, which have more planar intramolecular conformations exhibiting extended π conjugation, relative to the yellow polymorphs with their nearly perpendicular conformations that localize π electron density onto each ring.^126,138–140^ These systematic biases found for GGA and hybrid density functionals^140^ can be larger than the total experimental energy range spanned by the polymorphs.^195^

Fortunately, correcting the ROY intramolecular conformational energy contribution to the lattice energy using correlated wave function methods^126,211^ or density-corrected DFT^141^ dramatically improves the crystal energy rankings relative to experiment.^129^ As shown in Fig. 8, the resulting landscape reveals that the nine of the twelve lowest-energy candidate structures on the ROY landscape have already been crystallized experimentally. The four higher-energy forms (including the proposed-but-unconfirmed structure of the RPL polymorph^139^) are known to be metastable and/or were difficult to crystallize, suggesting that their less stable lattice energies are plausible. Interestingly, the calculations also suggest that the rank #15 structure on the landscape becomes the most stable form at high-pressure. While previous experimental high-pressure studies have not discovered any new polymorphs,^210,212^ this prediction suggests further efforts to produce high-pressure forms may be worthwhile.

Overall, the ROY system highlights how, despite many successful DFT-driven structure predictions, there are cases where the most frequently-used DFT-D functionals are inadequate. Similar problematic DFT delocalization error issues occur with conventional DFT functionals for the anti-cancer drugs galunisertib (Section 4.1) and axitinib,^126^ the photomechanical materials discussed in Section 4.5, and a number of other examples mentioned in Section 3. Fortunately, these errors can be overcome through intramolecular energy corrections of the sort used for ROY or the selection of a suitable density functional.^97^

### 4.4 Discovery of new porous organic materials

**Fig. 9:**
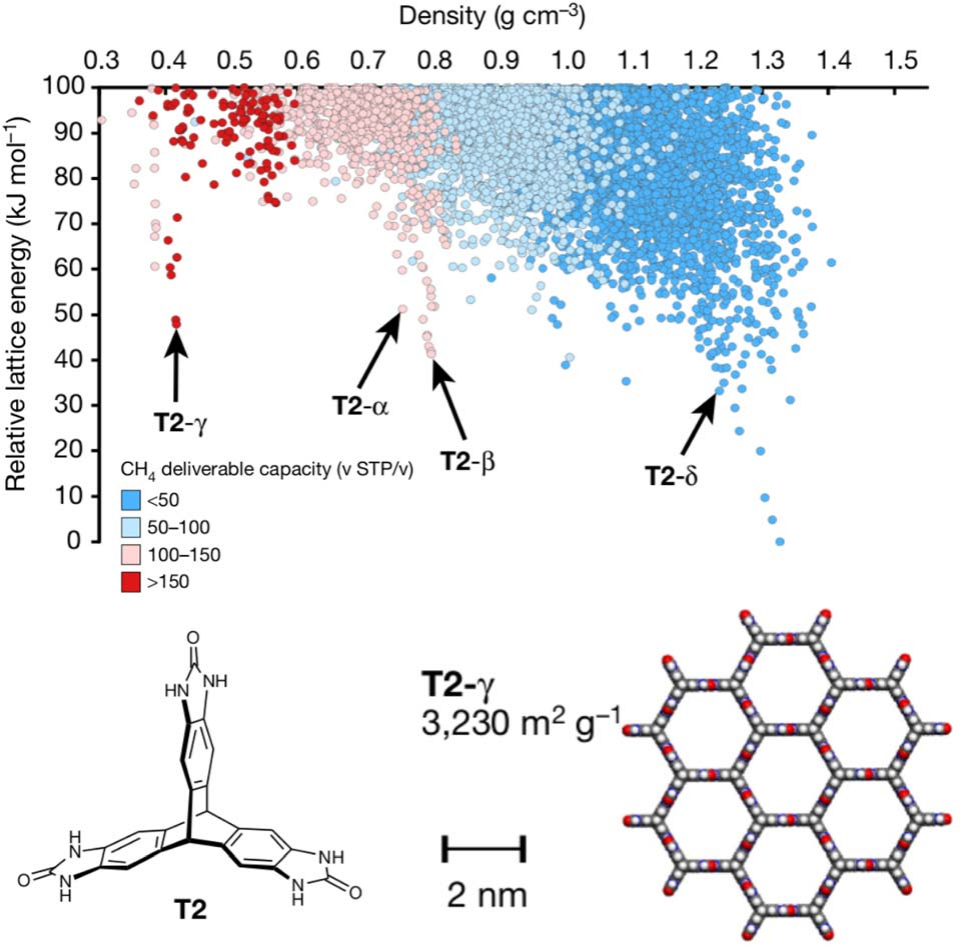
Crystal energy landscape for the molecule T2, color coded by the methane storage capacity. The α–δ polymorphs represent particularly stable porous crystals that have been crystallized experimentally. T2-γ set a record for the highest surface area among porous organic molecular crystals. Adapted with permission from ref. 213. Copyright 2017 Springer Nature.

Porous materials are useful for gas storage and separations, but rationally designing porous organic crystals is difficult. Beyond the usual difficulty in intuiting the relationship between molecular structure and crystal packing, porous organic crystals are exceptional because they counter the general thermodynamic preference toward dense crystal packing motifs. However, Pulido *et al.*^213^ demonstrated how CSP and energy-structure–function maps could be used to drive experimental discovery of new porous organic crystals. They began by performing CSP on a series of candidate molecular building blocks. As expected, the lowest-energy structures were densely packed and non-porous. However, they identified several interesting “spikes” higher on the crystal energy landscape which corresponded to unusually stable porous structures (Fig. 9). While these porous structures lay tens of kJ mol^−1^ above the global minimum—much too energetically unstable to crystallize on their own—the authors recognized that these putative porous structures would be dramatically stabilized by guest solvent molecules adsorbed within the pores.

Next, they computationally characterized the “function” of every structure on their predicted crystal energy landscapes—their methane storage capacity and their potential for hydrocarbon separations. From this combined understanding of a molecule's propensity to form porous structures and the resulting functional properties, they identified the molecule T2 (Fig. 9) as a promising candidate for new experimental crystallization screening studies. They discovered three new porous polymorphs of T2 in addition to a previously known one. The new *γ* form of this molecule set a record for ultra-high-surface-area organic materials (3425 m^2^ g^−1^). The experiments confirmed several predicted properties of these crystals, including surface area, gas storage capacities, and some gas separation properties. In select cases, however, adsorption of larger molecules during the gas separation testing induced experimental phase transitions that would have been difficult to anticipate computationally. Nevertheless, this study highlights how structure prediction and energy–structure–function maps can drive experimental discovery.^214^ Subsequent applications of these or similar concepts to porous materials^8,215–217^ and organic semi-conductors^218–221^ have further confirmed the role of CSP in the design and discovery of new organic materials.

While CSP often strives to accurately predict the most stable crystal structure using high-quality energy models, such detail is not always required to establish useful design principles for organic materials. Following on work that showed how the combined efforts of computation and experiments could lead to the rapid discovery of new porous organic cage materials for gas separation and storage applications,^222,223^ Wolpert and Jelfs demonstrated that a simple coarse-grained model could give meaningful insights into how porous organic cage molecules pack in the solid state.^224^ Rather than perform traditional, expensive CSP on each new large organic cage molecule, their simplified model represents the cages as octahedra with “patches” on each face that distinguish between whether they contain either an arene group or an open pore. After expressing the intermolecular interactions between patches *via* a simple model Hamiltonian, they explored the types of packing motifs that were preferred across a range of interaction parameter strengths. They developed a general understanding for how the chemical features of the cage molecules translate into the resulting crystal packing. Moreover, they can determine the key patch parameter values using just gas-phase dimer DFT-D calculations, and then predict the preferred crystal packing for a given species. The simplicity of this approach makes it highly amenable to high-throughput screening. Coarse-grained approaches like this can significantly narrow the molecular search space for new functional materials before performing more detailed CSP studies or experiments.

### 4.5 Establishing design principles for organic photomechanical engines

In organic photomechanical crystals, solid-state photochemical reactions can induce elongation, compression, twisting, bending, jumping, cracking, splitting, and other deformations of the crystal.^10–12^ Particular interest lies in harnessing these structural transformations to do mechanical work, such as for light-driven actuators^225–230^ or locomotion.^231,232^ Designing such materials requires understanding how molecular structure translates to crystal structure, how the crystal deforms due to the solid-state chemical reaction, and what anisotropic work is performed by that deformation. Characterizing these transformations experimentally is frequently challenging, since the solid-state chemical reactions may be incomplete, may be carried out in nanocrystals instead of bulk single crystals, and often produce short-lived and highly metastable polymorphs.

**Fig. 10:**
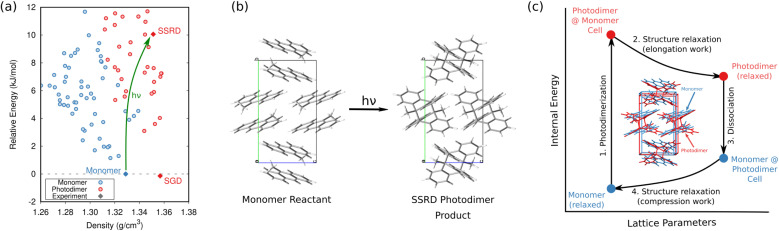
(a) CSP for the 9-methyl anthracene monomer reactant (blue) and photodimer product (red). The experimental monomer (solid blue) photochemically transforms to the solid-state reacted dimer (SSRD) polymorph, which differs from the solution-grown dimer (SGD) polymorph obtained from solution-phase crystallization. (b) Scheme showing the solid-state topochemical [4 + 4] photodimerization. (c) The photomechanical engine cycle involves fast photodimerization/dissociation reactions (Steps 1 & 3) followed by structural relaxations (Steps 2 & 4) which perform work. Figure adapted from ref. 135.

These systems therefore represent an excellent opportunity for first-principles structure prediction techniques. Until recently, the use of CSP in understanding solid-state reactions has been rare.^233^ We have now shown how CSP can be used as part of a strategy to predict or even design large photomechanical responses.^135^ The approach starts by predicting the crystal structures of the reactants and products (Fig. 10a). However, the bigger challenge lies in determining which of the many predicted product crystal structures is relevant. Because the solid-state reaction generates the product molecules within the crystal packing of the reactant, the product polymorph is typically thermodynamically metastable and often lies outside the typical ∼10 kJ mol^−1^ energy window associated with viable polymorph crystallization. Since CSP normally focuses on identifying the most stable crystal form(s), we instead apply topochemical principles to predict the solid-state transformation (Fig. 10b) and to identify the correct structure on the crystal energy landscape (Fig. 10a).

Once the structural transformations are known, the amount of work performed can be predicted. In the spirit of gas heat engines, we defined an idealized photomechanical engine cycle^135^ that enables computing the maximum work potentially performed by a given solid-state reaction (Fig. 10c). The engine model assumes the reaction occurs quickly and completely, thereby generating the product within the unit cell parameters of the reactant. Relaxation the crystal relieves the internal stress, deforms the crystal, and performs work.

Studies of solid-state [4 + 4] anthracene photodimerization^135,136^ and diarylethene ring opening and closing^137^ have revealed a number of important insights and design principles for organic photomechanical materials. First, the unique combination of high elastic modulus and large strains means that photomechanical organic crystals exhibit exceptional theoretical work densities up to at least 200 MJ m^−3^. If these could be realized experimentally, they would be several orders of magnitude larger than the work densities of elastomers or inorganic piezoelectrics. Second, the crystal packing proves crucial: for one diarylethene derivative studied, the maximum anisotropic work density differs 40-fold between two crystal forms. Based on the modest number of cases studied thus far, the range of work capacities across different polymorphs is broader than the range found for different photochromic species/reactions. This suggests that researchers should increase their emphasis on crystal engineering in selecting their target photochrome, especially since packing has a much larger impact on the mechanical response than do minor photochrome modifications (*e.g.* halogenation).^136^ Parallel alignments of the molecules in the crystal generally produce the especially large anisotropic deformations and work densities. Finally, the research has identified how the “memory” of the reactant crystal packing throughout the photochemical engine transformations biases them to produce greater work in the forward stroke direction than in the reverse one, enabling net work to be accomplished.

The need to understand solid-state chemical reactions extends beyond photomechanical systems. For example, solid-state photochemical degradation is a significant issue for pharmaceuticals, and crystal packing impacts photostability.^234^ Alternatively, crystal packing can be used to perform solid-state photochemical synthesis with precise stereochemistry and quantitative yields.^235–237^ Solid-state oxidations, reductions, isomerizations, bonds formations/cleavages, and many other reactions are also possible.^238^ Inducing solid-state reactions *via* mechanical grinding or milling (mechanochemistry) creates further synthetic possibilities.^239,240^ CSP can help engineer crystal packing motifs that either facilitate or inhibit solid-state reactions.

### 4.6 Rational design of organic semi-conductors

The ultimate promise of structure prediction lies in the complete rational design of new materials, starting from the molecular building blocks. Because the relationships between molecular structure and materials properties frequently cannot be intuited *a priori*, this rational design will likely require high-throughput crystal structure prediction to map these relationships across many candidate species.

True CSP-driven rational design of organic materials is still in its infancy, but Cheng *et al.* provided an intriguing peek at the future possibilities with their evolutionary exploration of organic semiconductor materials.^220^ Building on earlier work that had manually examined small numbers of species,^218,219^ they searched a chemical space of ∼68 000 aza-substituted pentacene isomers with (nearly) arbitrary connections between the five aromatic rings. Predicting crystal structures for every one of these molecules would be utterly impractical. Instead, they first screened candidate species using simple molecular-based fitness functions optimized *via* an evolutionary algorithm.

Next, crystal structure prediction was performed on the most promising molecular structures. In any medium- or high-throughput scenario, the models used to predict crystal structures will likely sacrifice some accuracy to achieve faster throughput. As a result, the predicted global minimum crystal structure for each species is less likely to correspond to the actual experimental crystal structures. To compensate, they evaluated the performance of each species by computing both the electron mobility of the most stable structure and a Boltzmann-weighted mean/standard deviation electron mobility across all low-energy candidate crystal structures. This latter metric identifies species whose crystal structures have consistently good mobilities, rather than those that happen to have a high mobility for one particular polymorph which may or may not occur experimentally.

**Fig. 11:**
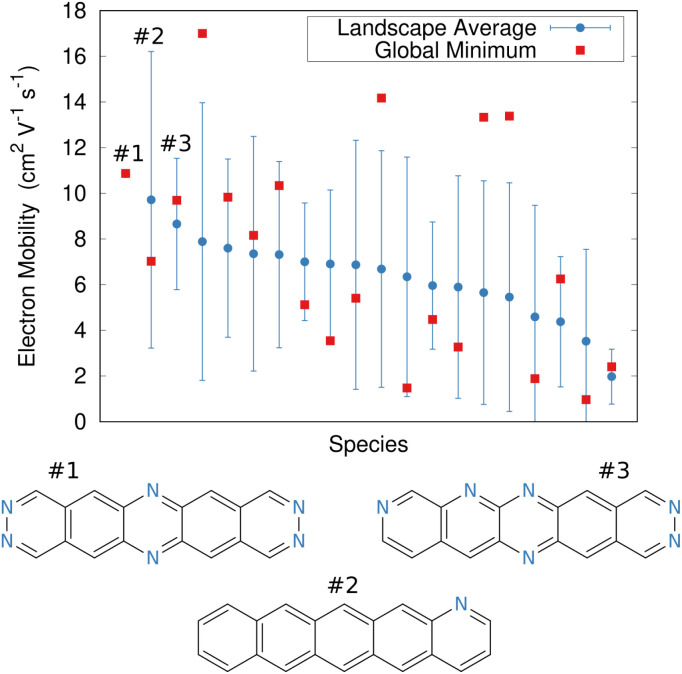
Predicted electron mobilities for various organic semi-conducting material candidates discovered through an evolutionary search.^220^ Red dots indicate the mobility for the most stable predicted structure of each species, while blue dots/error bars indicate the landscape-averaged mean mobility and standard deviation for the ensemble of low-energy structures. Three molecules with the highest landscape-averaged mobilities are shown.

In the end, the strong dependence of mobility on crystal packing meant that many candidates exhibited overlapping landscape-averaged mobilities and deviations (Fig. 11). On the other hand, the best candidate identified here proved to have only a single, low-energy structure which also had high mobility, thereby providing confidence that the predicted global minimum structure would likely match experiment. Overall, the evolutionary algorithm search identified candidates with good landscape-average and global minimum mobilities. The species identified by the evolutionary algorithm compared fairly well against several species that had previously been identified by human experts^241^ who used a mixture of computation and chemical intuition. Moving forward, incorporating solid-state properties directly into the molecular design search, rather than at the end, will likely to prove important for design of materials whose properties are very sensitive to crystal packing.

Separately, a CSP study correctly predicted the experimental crystal structure for chiral [6]helicene and rationalized the preference for the enantiopure crystal over the racemate.^221^ After this study demonstrated good but crystal packing-dependent semi-conducting properties, a follow-up CSP study then examined the impact of derivatizing [6]helicene molecules. Dimer screening was used to investigate over 1300 substituted helicenes, after which CSP was performed on the most promising candidates. In the end, they identified a set of derivatives which had predicted electron mobilities three times that of a previously characterized helicene.^242^

### 4.7 NMR crystallography

The combination of crystal structure prediction with spectroscopic experiments can be particularly helpful in solving challenging crystal structures. One can frequently identify the experimental crystal structure by predicting candidate crystal structures, simulating the relevant spectroscopic observables for each one, and comparing the results with experiment. While this general idea has been applied to various spectroscopic characterization experiments, including powder X-ray diffraction,^243–247^ Raman spectroscopy^153,248,249^ and transmission electron microscopy,^250^ nuclear magnetic resonance (NMR) crystallography represents the most widely-used combination.^39,251,252^

The combination of CSP, DFT chemical shift prediction, and solid-state NMR has been applied to many different pharmaceuticals and pharmacologically active compounds.^253–262^ One appealing feature of these approaches is that the spectroscopic observables provide a second metric for assessing candidate structures that is “orthogonal” to the lattice energy typically used in CSP. NMR crystallography can frequently solve the experimental crystal structure even when that structure is not the most stable one predicted by the CSP. Unsurprisingly, models which predict chemical shifts more accurately^263–266^ enhance the discrimination between correct and incorrect structural assignments in NMR crystallography.^267^ Further synergies between experiment and computation are also possible in NMR crystallography. For example, structural constraints inferred from the NMR experiments can accelerate the structure determination by reducing the size of the CSP search space.^258^

Machine learning models for predicting chemical shifts^268–284^ promise to accelerate NMR crystallography even further. The traditional NMR crystallography protocol involves first predicting a set of candidate crystal structures, computing the NMR spectra for each one, and comparing them against experiment, all of which can require substantial computational effort. Recently, however, Balodis *et al.* demonstrated how NMR crystallography can directly refine the crystal structure against the experimental solid-state NMR spectrum.^261^ The authors used Monte Carlo techniques to optimize an objective function which combined a weighted mixture of the structure's lattice energy and the error between its computed chemical shifts and experiment. These quantities can be evaluated inexpensively by using density functional tight binding (DFTB) for the energies and the machine-learning ShiftML model for the chemical shifts.^272,273^ Minimizing this objective function successfully produced the correct structures for several difficult crystals, despite the “moderate” accuracies of DFTB and ShiftML compared to DFT.

**Fig. 12:**
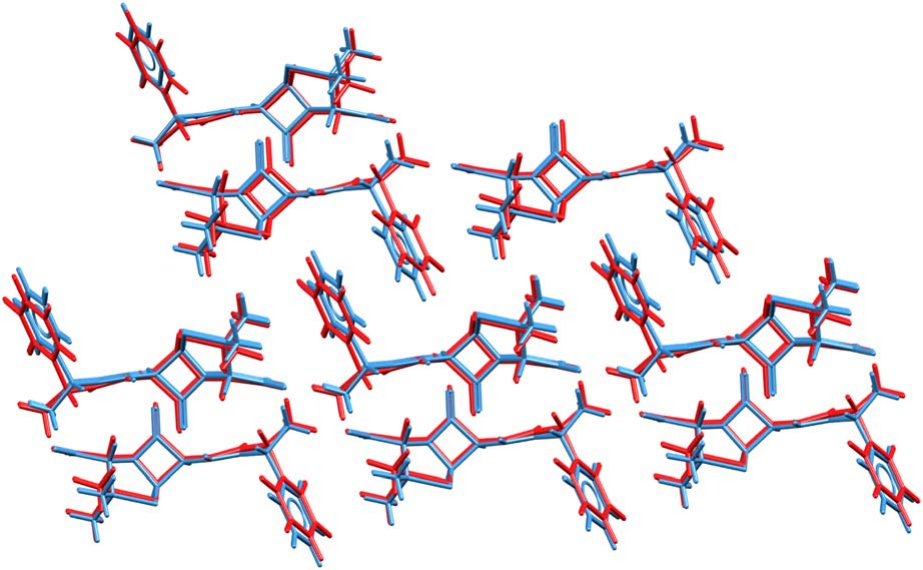
Overlay of the ampicillin crystal structures determined from X-ray diffraction experiments^285^ (blue) and the direct NMR crystallography solution (red) which employed a combination of DFTB energies and ShiftML chemical shifts.^261^

The ability to refine crystal structures directly from NMR will be especially beneficial for large, highly flexible molecules, for which traditional crystal structure prediction can be very expensive. For example, Balodis *et al.* solved the crystal structure of ampicillin (Fig. 12),^261^ despite the molecule adopting a high-energy intramolecular conformation in the solid-state that could easily be missed in a typical CSP search protocol. Separately, the rapid chemical shift prediction enabled by ShiftML also facilitated the structural characterization of amorphous drug candidate AZD5718.^260^ Advances like these are likely to significantly increase the effectiveness of NMR crystallography over the next few years.

### 4.8 Predicting solubility and mechanical properties

As crystal structure prediction becomes more routine, the focus will increasingly shift to computing the chemical and physical properties of the predicted crystals. In many cases, these properties will depend sensitively on the 3-D structure, making it important to account for the impacts of finite temperature on crystal structure and stability.^40^ Examples of computing properties related to gas separation and storage, photomechanical response, semi-conducting, and spectroscopy have already been discussed. Here, two more relevant properties are briefly discussed.

First, the solubility of organic species matters in many chemical applications, but it is especially significant in the pharmaceutical industry, where a large fraction of drugs in development suffer from low solubility. Because experimental measurements of solubility are time-consuming and resource-intensive, there has been considerable interest in predicting solubilities computationally. The field has frequently relied on informatics-driven approaches, including recent machine-learning efforts.^286–289^ Although such models can be very effective, they typically omit explicit solid-state contributions and therefore cannot account for how changes in crystal packing will impact solubility, for example.

There are on-going efforts to develop accurate physics-based models which could overcome this limitation. While some approaches simulate the solid–liquid coexistence directly,^290^ the more common approach employs a thermodynamic cycle that expresses the Gibbs free energy of dissolution from the free energies of sublimation and solvation,

4 Δ*G*_diss_ = Δ*G*_sub_ + Δ*G*_solv_ = −*RT* ln(*S*_0_*V*_m_)

The dissolution free energy can then be related to the solubility *S*_0_*via* the temperature *T*, molar volume of the crystal *V*_m_, and the ideal gas constant *R*. The sublimation free energy can be computed from periodic DFT calculations on the crystal lattice (and either approximating^291,292^ or explicitly computing the phonon contributions^293^) or MD simulations.^294^ The solvation free energy can be computed inexpensively *via* an implicit solvent model^292^ or more elaborately with explicit MD simulations. While developments in this space continue apace, increasing reliance on higher-quality quantum mechanical models in computing the free energy contributions is bringing the errors to accuracies that are already approaching the best informatics models.

Predicting relative solubility difference between two crystal forms is arguably easier, since that requires only the free energy difference between the two solid forms, avoiding the need to compute solvation free energies. For the drug rotigotine, for example, Mortazavi *et al.* predicted an 8.3-fold difference in solubility between the two polymorphs with DFT-D, in excellent agreement with the 8.1-fold difference measured experimentally.^172^

Second, knowledge of the mechanical properties of a material provides valuable insights into its durability and potential applications.^295^ Predicting the elastic constants enables one to screen materials *in silico* or to link features of the crystal packing to its mechanical response properties. For example, elastic constant predictions can be used to rationalize differences in how pharmaceuticals behave under tableting conditions. They helped explain the better tableting properties of paracetamol form II^296^ and several co-crystals^297^ compared to form I, of oxalic acid dihydrate over anhydrous oxalic acid,^298^ and of co-crystals of celecoxib.^299^ They showed that form II aspirin is mechanically stable,^300^ contrary to an earlier suggestion based on nano-indentation experiments that did not fully characterize the anisotropy of the crystal. Elastic calculations also helped confirm and rationalize the surprisingly large Young's moduli of amino acid crystals^301^ and a nucleic acid-based supramolecular assembly,^302^ and they gave insights into the negative linear compressibility in several organic acids.^303^ See a recent review for additional details.^295^

Early force-field predictions demonstrated the ability to compute elastic constants within ∼40–50% of experiment,^304^ with the neglect of thermal expansion of the crystal being a significant source of error. The accuracy with which these properties is predicted can be improved using quantum mechanical treatments and by accounting for thermal expansion (since mechanical properties are sensitive to molar volume). For example, the combination of accurate electronic structure methods and the quasi-harmonic approximation^143,305^ enables quantitative prediction of the mechanical properties of simple compounds such as carbon dioxide^147^ or deutero-ammonia.^93^ Such techniques are also quite effective in organic compounds such as urea,^145^ organic semi-conductors,^306^ and energetic materials.^307^ While DFT-D has become the most commonly-used approach, good-quality elastic constants can be obtained at lower computational cost. Spackman *et al.*^308^ curated a large data set of experimental elastic constants, demonstrated good consistency between experiment and the semi-empirical s-HF-3c model, and they even identified several suspicious experimental measurements based on large discrepancies between the experimental and computed results.

### 4.9 Can the predicted structures actually be crystallized?

The computational prediction of a new polymorph can sometimes drive its subsequent experimental discovery, as exemplified in the case of the cholesteryl ester transfer protein inhibitor drug Dalcetrapib.^27^ CSP on this drug correctly predicted the experimentally known form A and B polymorphs, which are closely related *via* a reversible temperature-dependent order-disorder phase transition. Interestingly, however, it also predicted another experimentally-unknown packing motif that lay very close in energy to form B. Motivated by lattice energy calculations that predicted this new structure to become more stable at pressures above ∼0.2 GPa, high-pressure crystallization experiments led to the discovery of a new form C. In the end, this new polymorph proved unstable at ambient conditions and is therefore unlikely to impact the pharmaceutical formulation. In this manner, CSP played an important role in managing the solid-form risks for this drug. Similarly, analysis of the CSP landscape and calculations of the free energies as a function of temperature and pressure led to the experimental discovery of a high-pressure polymorph of iproniazid.^171^

In a third example of CSP driving discovery, a study of the crystal energy landscapes of structurally-similar tolfenamic acid, mefanamic acid, and flufenamic acid identified many thermodynamically plausible isostructural crystal forms. Based on this analysis, the authors successfully templated two new thermodynamically metastable polymorphs of tolfenamic acid using crystals and solid solutions of the other species.^309^ Templating experiments informed by knowledge of predicted polymorphs similarly led to the discovery of new polymorphs of carbamazepine^310^ and cyheptamide,^311^ along with a new co-crystal of caffeine and benzoic acid.^312^

These cases all represent success stories for CSP, but there are counter-examples. The CSP-predicted global minimum energy crystal structure of galunisertib has never been found experimentally, despite years of effort.^26^ This is probably due to a combination of poor crystallization kinetics stemming from its very unfavorable intramolecular conformation^26^ and the fact that it is not actually the most stable structure (rather, it was artificially stabilized in the CSP by DFT delocalization error).^126^

More generally, identifying which putative CSP structures are likely to be crystallizable proves a major challenge. CSP usually predicts far more candidate crystal structures than are ever realized experimentally.^51^ For some of these structures, that simply means that the proper crystallization experiment has not yet been performed. More often, however, it reflects the limitations of CSP approaches, such as the focus on thermodynamic stability instead if kinetic crystallizability, the fact that many distinct lattice energy minima coalesce into a single free-energy basin at finite temperatures, and errors in the predicted energies (*e.g.* due to delocalization error biases). See ref. 51 for further discussion. Additional challenges in assessing synthesizablity and the challenges associated with theory-driven discovery of materials are discussed extensively in two recent reviews by Jelfs and co-workers.^32,33^

To improve our understanding of which predicted polymorphs will be experimentally relevant, on-going research efforts focus on reducing the number of crystal structures on the crystal energy landscape, on strategies for identifying crystals that are likely to be crystallizable, and on predicting the thermodynamic conditions under which different polymorphs are most likely to form. In 2005, Raiteri *et al.* demonstrated that metadynamics simulations dramatically simplified a benzene crystal energy landscape containing tens of lattice energy minima down to just a handful of structures on the free energy surface, most of which have been observed experimentally.^313^ The discarded structures are either labile, converting to different forms at finite temperature, or they correspond to different static structure representations from the same dynamic ensemble. Metadynamics has similarly been applied to reduce the crystal energy landscape of pigment red 179 (ref. 314) and, with less success, to 5-fluorouracil.^315^ Metadynamics and other enhanced sampling methods have proved effective for searching crystal energy landscapes directly,^313–322^ avoiding the need for post-hoc landscape reduction.

**Fig. 13:**
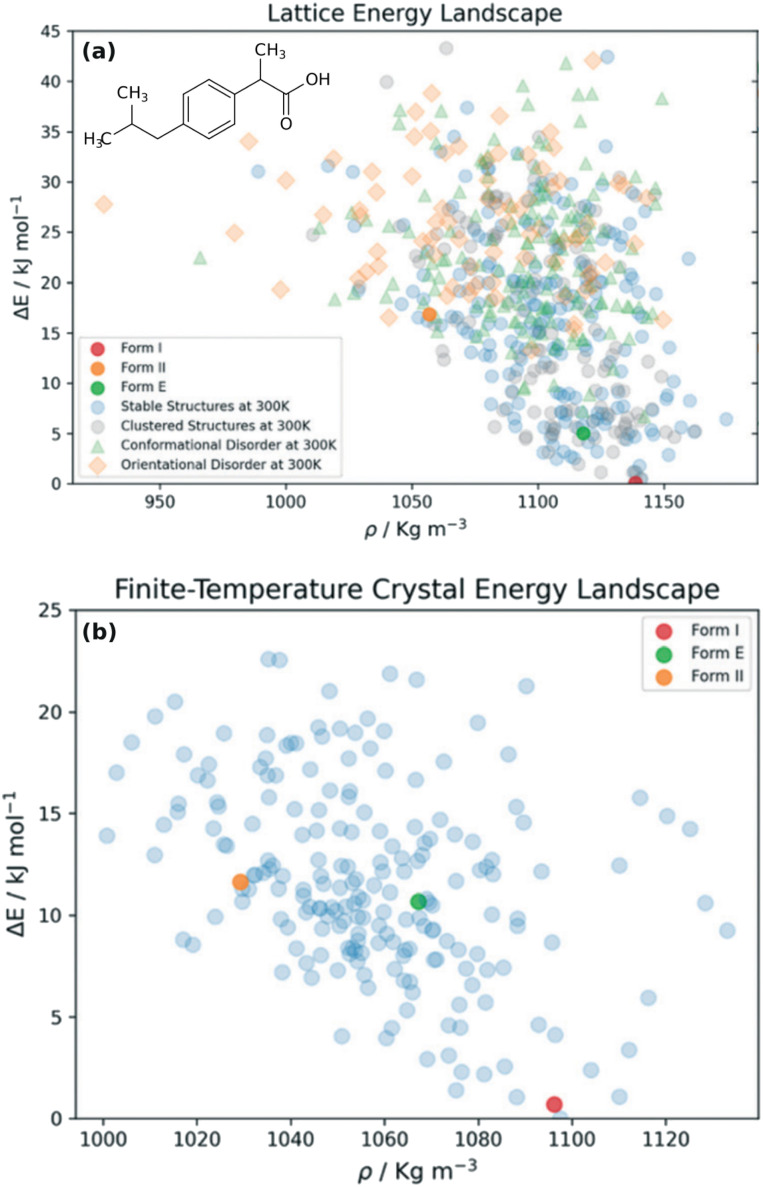
Elimination of (a) lattice energy minima that are labile or effectively equivalent at (b) finite temperatures using molecular dynamics, clustering, and enhanced sampling techniques reduces the number of predicted ibuprofen crystal structures by 65%. Figure adapted from ref. 323.

Recent efforts to systematize landscape reduction *via* a combination of MD, structure clustering, and metadynamics reduced the numbers of structures on the urea, succinic acid, and ibuprofen landscapes by ∼65–90%.^323,324^ The ibuprofen study is particularly impressive (Fig. 13), as its landscape containing 555 crystal structures is representative of the complexity of many real-world systems. Achieving this reduction has required significant efforts toward developing automated approaches for fingerprinting, simulating, and clustering the large numbers of structures involved.

The combination of CSP and metadynamics also helped rationalize the discrepant crystallization behaviors of two “sulflowers.” Experimentally, the original sulflower molecule crystallizes readily, while the structurally-related persulferated coronene forms only an amorphous solid.^325^ Using molecular dynamics and metadynamics, Sugden *et al.* demonstrated that while sulflower has a number of stable low-energy crystal forms (including the experimental crystal), all 20 of the lowest structures of persulferated coronene became disordered in the dynamics simulations, consistent with its amorphous behavior experimentally.^326^

A promising new, even simpler approach for landscape reduction based on the threshold method was demonstrated by Butler and Day.^327^ They coarsely estimate the energy barriers and structural relationships between predicted polymorphs *via* Monte Carlo moves that translate or rotate molecules and/or deform the unit cell parameter, accepting only moves that stay below a given energy threshold. In this manner, the approach identifies structures that can interconvert within a chosen energy threshold.^328^ A 5 kJ mol^−1^ threshold reduced the number of structures on the crystal landscape by ∼65–99% for several small organics.^327^

Landscape reduction does not guarantee that the remaining predicted crystal structures will be crystallizable. The understanding and modeling of nucleation and growth kinetics that leads to the crystallization of specific polymorphs remains difficult,^329^ though progress is being made.^52–55^ Instead, heuristic models are often used to identify crystal forms that are likely to crystallize. As noted in discussing galunisertib, conformational strain of the molecules in either the gas phase or appropriate solvents is sometimes considered as a factor in the crystallizabiliy of predicted polymorphs.^26,46,330,331^ Montis *et al.* estabilished a relationship between low surface roughness (rugosity) and crystallizability that can be used to infer the relative likelihood of crystallizing various polymorphs on the crystal energy landscape.^332^ In systems such as ROY, some of the less-stable polymorphs observed experimentally are among the smoothest, suggesting that kinetics favors their formation. In contrast, several other pharmaceutical polymorphs that have been difficult to produce have both higher energies and high rugocities, suggesting that both thermodynamics and kinetics hinder crystallization. The data-driven generalized convex hull approach of Anelli *et al.* is another promising strategy for exploring which crystal structures might be experimentally synthesizable.^333^

Finally, while predicting crystallization kinetics remains difficult, there has been progress in predicting the thermodynamic conditions under which a given polymorph will be preferred—*i.e.* polymorph phase diagrams. At pressures greater than ∼10–20 GPa, where factors such as thermal expansion become less significant, phase diagrams can be often be predicted with good accuracy.^152,334–338^ However, the situation is more difficult closer to ambient conditions, since the predicted phase transition temperatures can be extremely sensitive to small errors in the computed free energies.^339^ Despite these challenges, quite accurate temperature-dependent phase diagrams have been predicted for systems such as ice,^338^ carbon dioxide,^336,337^ methanol,^155^ and resorcinol.^156^ Polymorph phase transition temperatures have also been predicted for more complicated drug species.^30,48^ However, one should bear in mind how important fortuitous error cancellation is in predicting phase boundaries. For example, a 1 kJ mol^−1^ error in the relative free energies between α and β methanol alters the predicted ambient-pressure phase-transition temperature by more than 200 K!^155^

In the end, even if current CSP techniques cannot perfectly determine which crystal forms will be realized experimentally, they remain useful for assessing the polymorphic “risk” for a given species.^28,171^ This is particularly valuable for the pharmaceutical industry, as exemplified in the studies on galunisertib,^26^ gandotinib,^25^ hydrates,^340^ and salts^177^ discussed earlier.

## 5 Future outlook

Given the rapid developments in CSP over the last 5–10 years, it is interesting to speculate where new advances will occur over the next several years. First, it is likely that there will be increasing emphasis on using finite-temperature free energies instead of 0 K lattice energies for the final rankings. This is already routinely being done in the pharmaceutical industry, where compute budgets are typically larger than in academia, and it is sometimes done in academic studies as well. Rapid improvements in machine learning potentials will likely also increase the accuracy with which those free energy simulations can be performed by enabling dynamics-based approaches to be used on potentials that approach quantum mechanical accuracy.^44,101^ Improved understanding of how the strengths and weaknesses of widely-used DFT-D methods (*e.g.* delocalization and other systematic errors) impact crystal energetics will make it easier to identify when crystal energy rankings are likely to be problematic.

Second, interpretation of the crystal energy landscape will continue to gain in importance. Rapid developments in crystal energy landscape reduction are likely continue apace. Methods such as meta-dynamics or the threshold algorithm hopefully become much more widespread and routine. Once again, accurate, inexpensive potential energy models and structure clustering strategies based on ML should further improve the performance of these techniques.^44^ Improved uncertainty quantification for the computed structure energetics will also help users better assess the risks of predicted polymorphs on the landscape.

Third, it is not unusual for a high-accuracy crystal structure prediction to cost one million CPU hours per species at present. Entering the era of CSP-driven rational design will place a greater emphasis on performing “reasonably reliable” CSPs that have orders of magnitude lower computational cost, such that candidate materials can be screened en masse. Such approaches could mean learning to extract useful information from imperfect crystal energy landscapes (as in the organic semi-conductor design study discussed in Section 4.6), developing new intermediate ranking and refinement models (a.k.a. surrogate models^43^) that more effectively filter structures to reduce the number of final structures for which DFT-D calculations are needed, or even adopting entirely new data-driven topological approaches for generating short-lists of candidate structures quickly, without extensive hierarchical filtering algorithms.^44,341,342^

Fourth, beyond merely predicting structures, rational design efforts will increase the emphasis on computing functional properties of the putative crystals. Examples for gas storage and separations, organic semi-conducting properties, and photomechanical responses were already mentioned above. Feng and co-workers recently computed the photoluminescence properties of ROY and co-crystals of 9-acetylanthracene to understand the interplay of intra- and intermolecular interactions.^208^ Improved ability to predict pharmaceutical solubilties (Section 4.8) or to assess the photostability of candidate formulations (Section 4.5) would be very useful as well.

Fifth, as the applicability of CSP expands, there is also a clear need for the development of more user-friendly software tools to democratize access to CSP. Current CSP is still almost exclusively performed by specialists. In academic research environments, CSP often relies on a disjointed collection of software packages and home-built scripts for passing structures between them and processing the results. CSP tools developed in industry are more user-friendly, though those companies often cater more to larger budgets and computing capabilities of pharmaceutical companies than to smaller-scale academic research groups. Moreover, new method developments from different groups are not always widely/publicly available in the short-term. Of course, radical reductions in computational cost will also be needed to enable truly widespread use of CSP by non-expert practitioners.

## 6 Conclusions

In conclusion, crystal structure prediction has advanced dramatically to the point where experimental crystal structures can be predicted successfully much more often than not. Applications of CSP have moved on from small-molecule benchmarks to real-world pharmaceutical formulations and functional organic materials. New frontiers are opening in areas such as the ability to use CSP to rationally design new materials with targetted properties or to model solid state chemical transformations. Identifying which predicted crystal structures can be made experimentally has been challenging, though good progress is being made there as well. Heinlein's dream of theory-driven materials design is quickly becoming reality, even if it is a couple decades late.

## Author contributions

G. J. O. B. conceived and wrote this article.

## Conflicts of interest

There are no conflicts to declare.

## Supplementary Material
